# Identifying dominant modifiers of mutant FUS toxicity *in vivo*

**DOI:** 10.1186/1750-1326-8-S1-O33

**Published:** 2013-09-13

**Authors:** J Gavin Daigle, Nicholas A Lanson, Ian Casci, John Monaghan, Charles Nichols, Dimitri Kryndushkin, Frank Shewmaker, Udai Pandey

**Affiliations:** 1 Department of Genetics, Louisiana State University Health Sciences Center, New Orleans, LA, USA; 2 Department of Pharmacology and Experiment Experimental Therapeutics, Louisiana State University Health Sciences Center, New Orleans, LA, USA; 3 Department of Pharmacology, Uniformed Services University of the Health Sciences, Bethesda, MD, USA

## 

FUS is a DNA/RNA binding protein found to be mutated in some cases of both sporadic and familial forms of ALS. It is still not clear how ALS-causing mutations in FUS leads to motor neuron degeneration. Here, we exploited a Drosophila model and mammalian neuronal cell lines to elucidate the role of the RNA-binding ability of FUS in regulating FUS-mediated toxicity. To determine the role of the RNA binding ability of FUS in ALS, we mutated FUS RNA binding sites (F305, F341, F359, F368) to leucines and generated RNA binding-incompetent mutants (4F-L) with and without ALS causing mutations R518K or R521C. We found that mutating 4F to L residues makes FUS RNA binding-incompetent. We observed that ectopic expression of RNA binding-incompetent FUS in fly brain, eyes and motor neurons strongly blocks neurodegenerative phenotypes as compared to RNA-binding-competent FUS carrying ALS causing mutations. Interestingly, RNA-binding deficient FUS strongly localized to the nucleus of Drosophila motor neurons and mammalian neuronal cells whereas FUS carrying ALS linked mutations was distributed to the nucleus and cytoplasm.

Importantly, we found that incorporation of mutant FUS into stress granules is dependent on the RNA-binding ability of FUS. SGs are dynamic aggregates composed of proteins and RNA that are formed when cells are under a variety of stresses. We observed that normally cytoplasmic SGs rapidly disassemble when stress conditions end, whereas cytoplasmic SGs formed in ALS patient cells having a FUS mutation fail to disassemble. This suggests that mutant FUS sequesters proteins and RNAs important for cellular homeostasis and the defect in disassembly of cytoplasmic SGs contributes to ALS. Finally, following up these observations we performed an *in vivo* unbiased genetic screen that led to the discovery of several dominant modifiers of mutant FUS toxicity including proteins involved in regulating SG dynamics, mitochondrial functions and RNA splicing. These genetic modifiers would help in identifying potential therapeutic targets for ALS.

